# Case Report: Hematopoietic Stem Cell Transplantation to Treat Severe Acquired Aplastic Anemia in a Pediatric Kidney Transplant Recipient

**DOI:** 10.1111/petr.70108

**Published:** 2025-05-30

**Authors:** Gintarė Mierkienė, Goda Elizabeta Vaitkevičienė, Karolis Ažukaitis, Augustina Jankauskienė, Jelena Rascon

**Affiliations:** ^1^ Clinic for Children's Diseases, Institute of Clinical Medicine, Faculty of Medicine Vilnius University Vilnius Lithuania

**Keywords:** children, hematopoietic stem cell transplantation, kidney transplantation, severe aplastic anemia, transplantation‐associated thrombotic microangiopathy

## Abstract

**Background:**

Hematopoietic stem cell transplantation (HSCT) in solid organ transplant recipients has been reported in adults. However, data on children are scarce. We report a case of an allogeneic HSCT in a 14‐year‐old girl to treat idiopathic very severe aplastic anemia (SAA).

**Case Presentation:**

The girl developed end‐stage renal disease at the age of 4 years following Shiga toxin hemolytic‐uremic syndrome. The cadaveric kidney was grafted at the age of 7 years. Three years later, the patient was successfully treated for active humoral graft rejection and continued with tacrolimus and antihypertensive treatment. At 10 years, an absence epilepsy manifested; therefore, lamotrigine and ethosuximide were added. After 7 years of having a kidney transplant, the patient developed very severe pancytopenia and was diagnosed with SAA. Parvovirus B19 and EBV infections were documented. At the age of 14 years, she received allogeneic hematopoietic stem cells from a matched unrelated CMV‐seronegative donor. Neutrophils engrafted on Day +19 and full donor chimerism was achieved. An acute graft‐versus‐host disease grade II regressed after the escalation of immune suppression, which aggravated arterial hypertension and triggered CMV reactivation treated with glomerular filtration rate‐adjusted ganciclovir. Antiviral therapy deteriorated renal graft function. A high‐risk transplantation‐associated thrombotic microangiopathy was diagnosed on Day +42 and treated with eculizumab. Despite adoptive therapy with CMV‐specific cytotoxic T‐lymphocytes (Day +62) the pericardial effusion developed and required surgical drainage. Nevertheless, CMV viremia and polyserositis gradually progressed to multiorgan failure. The patient died on Day +95 after HSCT.

**Conclusions:**

Despite reported favorable outcomes in children who received allogeneic HSCT after kidney transplantation, there is a lack of evidence on how to overcome numerous challenges in these ultrarare cases.

**Abbreviations**
AKI: acute kidney injury
ATG: anti‐thymocyte globulin
CMV: cytomegalovirus
CSA: cyclosporin A
DNA: deoxyribonucleic acid
EBV: Epstein‐–Barr virus
eGFR: estimated glomerular filtration rate
GvHD: graft‐versus‐host disease
HSCT: hematopoietic stem cell transplantation
IST: immunosuppressive therapy
MMF: mycophenolate mofetil
MUD: matched unrelated donor
PD: peritoneal dialysis
SAA: severe aplastic anemia
SOT: solid organ transplantation
TA‐TAM: transplantation‐associated thrombotic microangiopathy

## 1 Introduction

There is growing evidence supporting the feasibility of sequential hematopoietic stem cell transplantation (HSCT) in solid organ transplant recipients [1, 2, 3, 4]. Most reports come from adults; however, data on children are scarce. Significantly lower transplant activities in childhood might underpin this: overall, the most common hematopoietic stem cell graft volume is 10 times lower in children than in adults [5].

Allogeneic HSCT is a standard procedure to treat acquired severe aplastic anemia (SAA) in children, reaching a 5‐year overall survival of over 90% [6, 7]. In rare cases, when allogeneic stem cells were offered to adult solid organ graft recipients to revert irreversible damage to hematopoiesis or overcome hematologic malignancies, the 5‐year overall survival did not exceed 42%–46% [1, 2, 8]. The results appear to be affected by the type of solid organ transplantation (SOT) with more favorable outcomes in patients after liver rather than kidney transplantation [1, 2, 4, 8, 9]. A trend toward a higher cumulative incidence of solid organ graft failure in the kidney as compared to liver transplant recipients was reported [8]. Shinohara et al. described a significantly lower kidney function 1 year after HSCT than before in five out of nine adults who received allogeneic hematopoietic stem cells after kidney transplantation, with seven out of nine patients finally deceased [1].

HSCT is rarely used in children after SOT. The data are limited to case reports or include minors in joint adult‐pediatric cohorts. When HSCT was performed following SOT, the liver was the most frequently transplanted solid organ [2, 8, 10, 11]. Case reports describing HSCT in pediatric heart or kidney recipients are even more scarce [8, 10, 12]. Likewise, in adults, the outcomes seem to be rather favorable in liver recipients—based on the available data, 15 out of 22 children were alive after HSCT [2, 8, 10, 11, 13]. Three out of four children who received allogeneic stem cells following heart transplantation did not survive [8, 10, 12], whereas two pediatric kidney and stem cell recipients were alive at the time of publication [8]. Herein, we aim to share our experience in the management of a challenging case of allogeneic HSCT to treat SAA in a 14‐year‐old kidney transplant recipient.

## 2 Case Description

**FIGURE 1:**
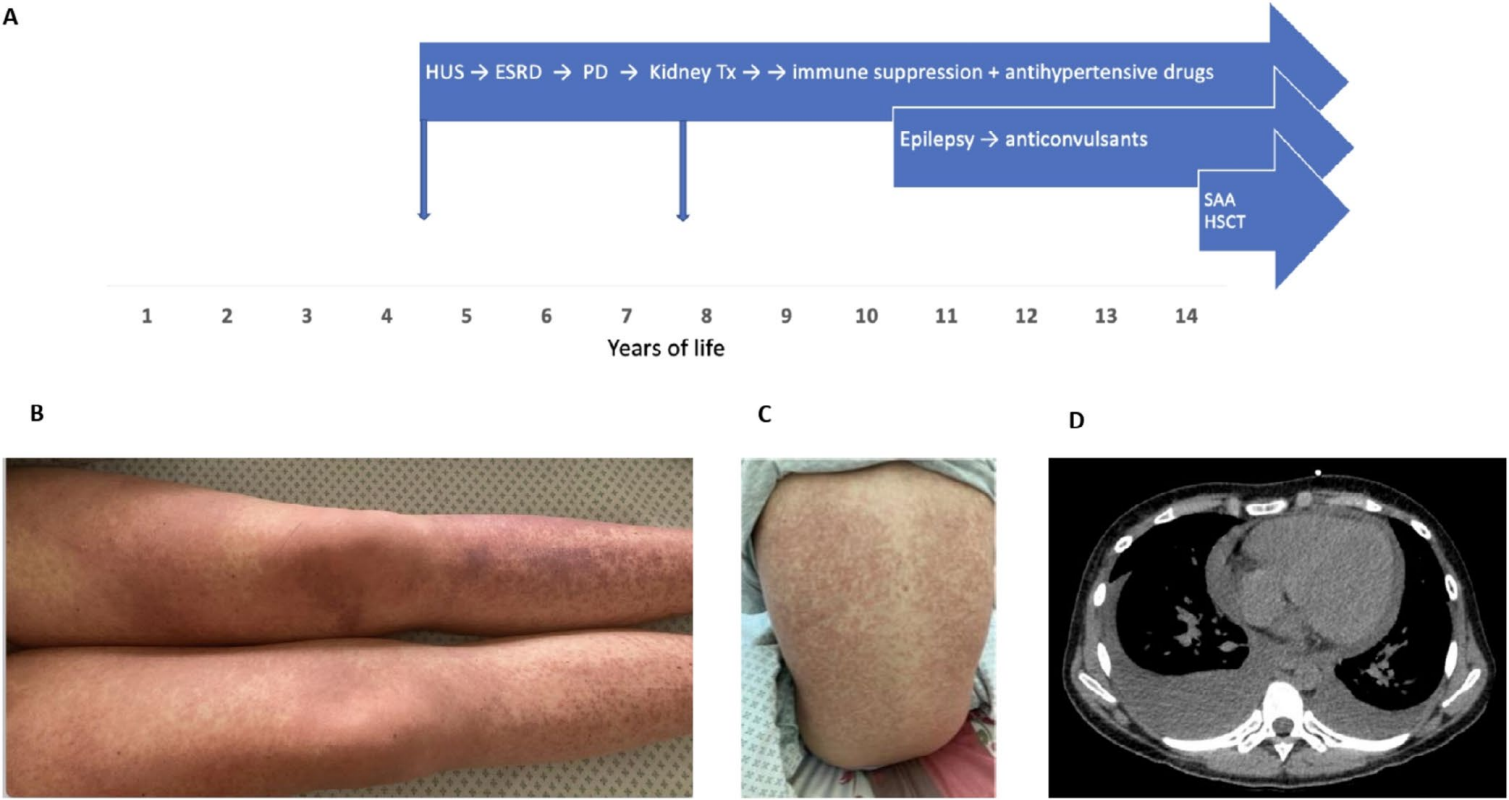
Clinical course. (A) Disease burden through the life span. (B) Rash 1 month before HSCT, considered as an allergy to itraconazole and/or lamotrigine. (C) Skin acute GvHD after allogeneic HSCT (Day +40). (D) Pleural and pericardial effusion (Day +68). ESRD, end‐stage renal disease; HSCT, hematopoietic stem cell transplantation; HUS, hemolytic uremic syndrome; PD, peritoneal dialysis; SAA, severe aplastic anemia; Tx, transplantation.

A previously healthy girl born from non‐consanguineous parents without a known family history of kidney or hematologic disease was diagnosed with Shiga‐toxin *Escherichia coli* hemolytic uremic syndrome at the age of 4 years (Figure 1A). The patient developed anuric acute kidney injury (AKI) and required peritoneal dialysis (PD) for several months. Her kidney function did not fully recover, and she gradually developed Stage 5 chronic kidney disease and was eventually started on automated PD at the age of 6 years. Her course on automated PD was uneventful except for periodically appearing spontaneous ecchymoses on the legs that were attributed to a mild uremia‐associated thrombocytopathy (no thrombocytopenia was observed). At the age of 7 years and 8 months, the patient received a cadaveric kidney transplant (3 mismatches, 3% of panel‐reactive antibodies, cytomegalovirus (CMV) status donor+/recipient–, induction with basiliximab). The immunosuppressive regimen consisted of tacrolimus and mycophenolate mofetil (MMF). Persistent otherwise unexplainable leukopenia was observed, and 3 years after the kidney transplantation, MMF was discontinued. White blood cell count increased but remained fluctuating within the interval of 1.5–3.0 (10^9^/L). The patient continued on tacrolimus monotherapy (trough levels between 3.0 and 3.96 ng/mL). At the age of 13 years, 6 years after kidney transplantation, proteinuria (1.5 g/L) was noticed while the estimated glomerular filtration rate (eGFR) was normal (94 mL/min/1.73 m^2^). A transplant biopsy revealed active humoral graft rejection with peritubular leucostasis (ptc2) and diffuse C4d deposition in peritubular capillaries (c4d3). The patient received treatment with high‐dose methylprednisolone pulse therapy (a total of three pulses of 2000 g). Proteinuria decreased from 1.5 to 0.75 g/L, and the patient continued on tacrolimus) and two antihypertensive medications. Three months after the diagnosis of rejection, the patient developed absence epilepsy, which was controlled with lamotrigine and ethosuximide. Soon after the diagnosis of epilepsy, the patient developed severe headaches, and migraine was diagnosed that required intermittent treatment with sumatriptan.

A year later, at the age of 14 years and 2 months, the girl had a mild sore throat episode, managed at home. Her family had a SARS‐CoV‐19 infection confirmed by a rapid antigen test at home. One month after the presumed infection episode, the patient was hospitalized with a 3‐day history of persistent febrile fever, nausea, weakness, sore throat, mild cough, and prolonged metrorrhagia. Physical examination revealed pharyngeal and tonsillar hyperemia with a plaque on the right tonsil, normotension (120/69 mmHg), and tachycardia (120 bpm). Her complete blood count revealed severe pancytopenia with normal eGFR and tacrolimus level of 3.5 ng/mL (Table 1). CRP was elevated however respiratory viral and bacterial work‐up did not reveal any infectious trigger. Laboratory values at the time of admission are summarized in Table 1. Diagnostic work‐up for possible underlying infection ruled out influenza, active SARS‐CoV‐2 and CMV infections. Empiric therapy with wide‐spectrum antibacterial agent ceftazidime for febrile neutropenia was started which was later switched to meropenem and antifungal therapy with fluconazole was added. Following the positive Parvovirus B19 PCR tests, a course of intravenous immune globulin (cumulative dose 2 g/kg) was administered considering possible Parvovirus B19‐induced anemia and immune thrombocytopenia without improvement of pancytopenia. The condition was complicated by protracted and painful metrorrhagia, requiring massive transfusion support with packed red blood cells and platelet concentrates (the coagulation panel was normal), pain killers, and was finally managed by administering oral contraceptives (ethinylestradiol and dienogest). The dose of ethosuximide was decreased considering potential drug‐induced myelotoxicity, and the dose of lamotrigine was increased to maintain the anticonvulsant effect.

**TABLE 1:** Laboratory values at the time of admission.

	Patient's value	Institutional reference range
Blood count		
White blood cells (×10^9^/L)	1.24	4.0–10.0
Neutrophils (×10^9^/L)	0.02	1.5–6.0
Platelets (×10^9^/L)	21	140–450
Hemoglobin (g/L)	90	120–145
Lymphocytes (×10^9^/L)	1.06	1.0–4.0
Reticulocytes (×10^12^/L)	0.006	0.9–1.5
Biochemistry		
CRP (mg/L)	133	< 5
Creatinine (μmol/L)	84	< 68
eGFR (mL/min/1.73 m^2^)	58	> = 90
Ferritin (μg/L)	285	7–140
D‐dimers (μg/L)	625	80–195
Fibrinogen (g/L)	4.02	2–4
LDH (U/L)	151	< 279
Triglycerides (mmoL/L)	2,21	(1, 3)
Soluble CD25 (IL‐2Ra) (pg/mL)	4287 ± 10	890–1035
Infection work‐up		
Combined rapid SARS‐CoV‐2/Influenza A/Influenza B antigen test	negative	negative
SARS‐CoV‐2 IgG antibodies (BAU/mL)^a^	603.7	negative
Respiratory infection viral PCR panel^b^	negative	negative
Respiratory infection bacterial PCR panel^c^	negative	negative
Parvovirus B19 DNA in blood (copies/mL)	positive Ct 39.17	negative
Parvovirus B19 DNA in bone marrow (copies/mL)	negative	negative
CMV virus DNA in blood copies/mL)	negative	negative
EBV virus DNA in blood (copies/mL)	1250	negative
EBV virus DNA in bone marrow (copies/mL)	10 600	negative
Polyoma BK virus DNA in urine (copies/mL)	5550	negative
Polyoma BK virus DNA in blood (copies/mL)	negative	negative
Herpes 6 virus DNA in blood (copies/mL)	negative	negative
Herpes 8 virus DNA in blood (copies/mL)	negative	negative
Herpes 7 virus DNA in blood (copies/mL)	negative	negative
Blood culture	negative	negative
Urine		
Proteinuria (g/L)	0.75	negative
Hematuria (RBC/μL)	250	negative
Immunology		
Total lymphocyte count (cells/μL)	1508	2000–3700
CD3+ (cells/μL)	1282	1400–2000
CD19+ (cells/μL)	166	200–700
CD16/CD56+ (cells/μL)	15	200–400

Abbreviations: BAU, binding antibody units; CMV, cytomegalovirus; CRP, C‐reactive protein, Ct, cycle threshold; DNA, deoxyribonucleic acid; EBV, Epstein–Barr virus; eGFR, estimated glomerular filtration rate; IgG, immune globulin G; LDH, lactate dehydrogenase; PCR, polymerase chain reaction; polyoma BK, human polyomavirus 1; RBC, red blood cells; SARS‐CoV‐2, severe acute respiratory syndrome coronavirus 2.

^a^ SARS‐CoV‐2 positive PCR documented 1 year before the current admission.

^b^ Respiratory infection viral PCR panel included adenovirus, influenza B, parainfluenza virus 1–4, rhinovirus A/B/C, respiratory syncytial virus A and B, bocavirus 1/2/3/4, coronavirus 229E, NL63, and OC43, metapneumovirus, enterovirus.

^c^ Respiratory infection bacterial PCR panel included *Chlamydophila pneumoniae, Mycoplasma pneumoniae, Bordetella pertussis, Bordetella parapertussis, Legionella pneumophila, Streptococcus pneumoniae*, and *Haemophilus influenzae* .

**FIGURE 2:**
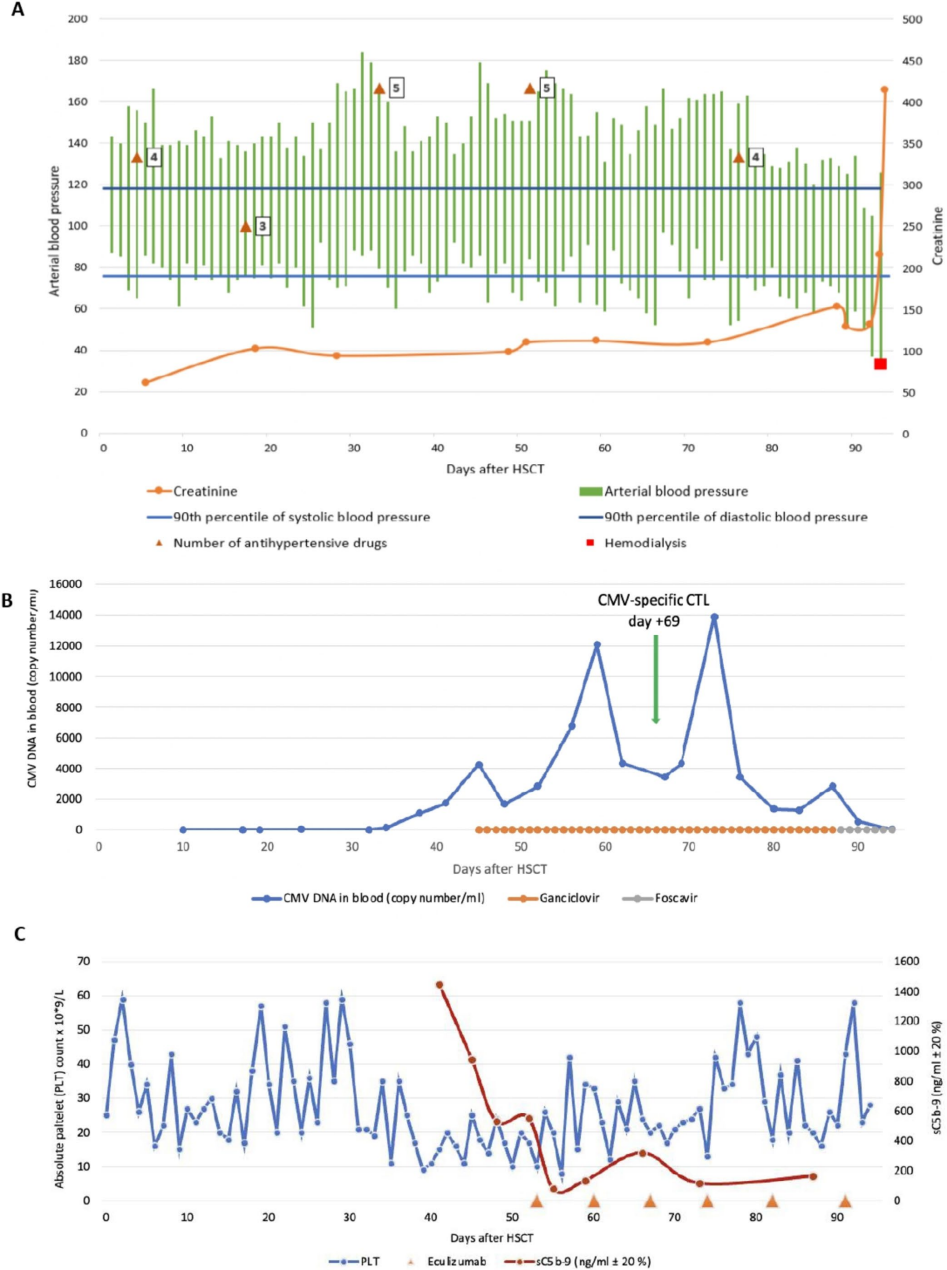
Post‐transplant course. (A) Arterial blood pressure. (B) Cytomegalovirus reactivation and its treatment. (C) Platelet count and sC5b‐9 change on eculizumab treatment.

Bone marrow aspirate and trephine biopsy revealed severe pancytopenia without myelodysplastic features and normal karyotype consistent with SAA. The diagnosis of SAA was confirmed at the pediatric EWOG‐MDS reference center in Freiburg. EBV‐DNA (10 600 copies/ml) but no Parvovirus B19 was found in bone marrow. The allogeneic HSCT procedure was initiated as the standard first‐line therapy for SAA in our center, taking into account the prolonged immunosuppression treatment already received. No sibling donor was available and a decision to infuse stem cells from an unrelated donor was made. Tacrolimus was switched to cyclosporin A (CSA) during the donor search. Several episodes of febrile neutropenia were managed with broad‐spectrum antibacterial (ceftazidime, piperacillin with tazobactam, meropenem) and antifungal (itraconazole) therapy. A purpura skin rash appeared after the start of itraconazole (Figure 1B) and an allergic reaction to itraconazole and/or lamotrigine was considered. The rash improved gradually after initiation of methylprednisolone 1 mg/kg. Peripheral stem cells (6.6 x 10^6^ CD34/kg, 4.01 x 10^8^ CD3/kg) from a CMV‐seronegative, ABO‐compatible, and 9/10‐matched unrelated donor (MUD) were infused after fludarabine, cyclophosphamide, and alemtuzumab conditioning. Graft‐versus‐host disease (GvHD) prophylaxis with CSA (targeting the level of 150 ng/mL) and methotrexate was administered. The preparative regimen did not cause any unexpected toxicity except for an episode of seizures that required a 24‐h stay at the intensive care unit. The patient was continued with three anticonvulsants (levetiracetam, ethosuximide and lamotrigine). A systemic bacterial infection before engraftment was managed with meropenem with concomitant antifungal prophylaxis using caspofungin. Neutrophil engraftment was documented on Day +19; however, the patient remained platelet transfusion dependent. Complete donor chimerism (donor DNA > 99% in whole blood cells and CD3, CD19, and CD15 cell populations) was documented on Day +28 after HSCT and remained stable throughout the post‐transplantation period. During the peritransplant period, kidney transplant function was normal (eGFR > 90 mL/min/1.73 m^2^), however, continuous therapy with multiple antihypertensive medications was required to control severe arterial hypertension (BP > 99th percentile) (Figure 2A).

Local facial hyperemia on Day +21 was interpreted as acute GvHD and treated with topical steroids. However, the rash spread all over the body and immune suppression was escalated by increasing the CSA dose and adding MMF. The skin biopsy revealed lymphocytic vasculitis compatible with drugs or viral infection‐driven inflammation, but not GvHD. Finally, on Day +38, the patient was clinically diagnosed with GvHD grade II (Figure 1C) Gastrointestinal involvement manifested as vomiting and loss of appetite. No GvHD‐related liver injury appeared at any time. Methylprednisolone 1 mg/kg/day was added to CSA and aggravated markedly pre‐existing hypertension, which required intensification of antihypertensive treatment from 3 to 5 drugs (Figure 2A). The increase in CSA dose deteriorated kidney function (1.5‐fold increase in serum creatinine). To alleviate GvHD and prevent escalation of immune suppression, two doses of mesenchymal stem cells were infused on Day +45 and +52, respectively. The intensified immune suppression improved cutaneous GvHD but triggered reactivation of CMV and BK virus. Intravenous GFR‐adjusted ganciclovir twice daily was initiated on Day +45. After initial response, CMV reactivation reflared; therefore, given insufficient immune reconstitution (Table S1), CMV‐specific cytotoxic T lymphocytes (CTL) were infused on Day +69. However, despite these efforts, CMV viremia increased. The change in CMV copy load is depicted in Figure 2B.

On Days +40 to +42, transplantion‐associated thrombotic microangiopathy (TA‐TMA) was suspected due to transfusion‐resistant thrombocytopenia, severe arterial hypertension, and worsening of proteinuria and anemia. The focused investigation revealed elevated sC5b‐9, decreased haptoglobin, schistocytes in the blood smear, and a negative Coombs's test, summarized in Table 2. The patient was diagnosed with TA‐TMA and treated with weekly intravenous eculizumab (900 mg). A total of six doses of eculizumab were administered. Following eculizumab administration, spontaneous platelet count recovery occurred, and the patient became transfusion independent, with platelet engraftment documented on Day +73 (Figure 2C).

**TABLE 2:** Transplantation‐associated thrombotic microangiopathy (TA‐TMA) diagnostic criteria as per Harmonization Panel Consensus Recommended Diagnostic Criteria [14]. Modified Jodele Criteria and patient's corresponding values (present features are in bold).

TMA diagnostic criteria	Age adjusted institutional reference range	Days after HSCT			
		+41	+45	+51	+61
Thrombocytopenia (*10 ^**9**^ **/L)**	140–450	15	11	20	23
**Anemia Hgb (g/L)**	120–145	94	87	80	93
**Schistocytes score in blood smear**	0–3	1	1	0	0
**Elevated sC5b‐9 (ng/mL)**	200–325	1445% ± 20%	944% ± 20%	553% ± 20%	136% ± 20%
**Proteinuria (g/L)**	< 0.1	5	1.5	0.75	0.75
Elevated LDH (U/L)	< 279	220	193	180	162
**Arterial hypertension (mmHg, 99 percentile for 13‐year‐old female)**	≥ 130/80	148/111	153/99	151/101	137/96
Additional tests					
Decreased haptoglobin (g/L)	0.3–2.0	< 0.08	< 0.08	< 0.08	< 0.08
ADAMTS‐13 (IU/mL)	0.40–1.50	0.86% ± 20%	n. a.	n. a.	n. a.
Coomb's test	Negative	Negative	n. a.	n. a.	n. a.
CH50 pg. eq/mL	55–75	112% ± 10%	n. a.	n. a.	n. a.

Abbreviations: ADAMTS‐13, a disintegrin‐like and metalloprotease with thrombospondin type 1 repeats; CH50, The total complement activity assay; HSCT, hematopoietic stem cell transplantation; Hgb, hemoglobin; IgG, immune globulin G; LDH, lactate dehydrogenase; n. a., not assessed; sC5b‐9, soluble terminal complement complex.

On Day +66, the patient developed periorbital edema and dry cough. Chest computed tomography revealed bilateral pleural and pericardial effusion (Figure 1D) and inflammatory infiltration in both lungs. Despite intensified intravenous diuretics, echocardiography after a week showed deterioration of diastolic function. On Day +77, echocardiography revealed severe pericardial effusion (28 mm at maximum measurements) and a right pericardial and pleural window was formed along with continuous drainage of the pleural cavity. Fifty copies/ml of CMV were found in the pericardial fluid. The pericardial biopsy revealed signs compatible with chronic fibrosing pericarditis. The patient was transferred to the PICU; however, her condition continued to deteriorate gradually due to increasing respiratory failure, while her heart function remained stable. Pleuropericardial drainage (including the need to drain the right pleural cavity) was continued daily, yielding from 50 to 600 mL exudate. Antiviral, antibacterial, and antifungal therapy was continued along with eculizumab; however, on Day +90, the condition worsened. A lung ultrasound showed bilateral pulmonary edema and consolidation in the right lung. CMV DNA in blood increased from 1270 to 2813 copies/ml. Due to decompensated respiratory failure, mechanical ventilation was started. Bronchoalveolar lavage revealed 3015 copies of CMV‐DNA. Ganciclovir was switched to foscavir (Figure 2B). The patient then became hypotensive, requiring noradrenaline infusion (Figure 2A). Anuric AKI developed soon after, and continuous venovenous hemodiafiltration was started on Day +93. Despite all efforts, the patient died on Day +95 after allo‐HSCT, being 14 years and 9 months of age.

## 3 Discussion

This case report describes an adolescent who developed SAA 7 years after kidney transplantation, eventually requiring allogeneic HSCT. The case of this patient reflects on the many challenges that may be encountered in patients developing SAA and requiring allo‐HSCT following kidney transplantation. Kidney transplant recipients are at increased risk of developing diverse conditions that may manifest as peripheral blood cytopenias, either of isolated cell lines (anemia, leukopenia, and thrombocytopenia) or involving all three cell lines. The causes of those most frequently include medications (calcineurin inhibitors, mycophenolic acid, valganciclovir, and others) and viral infections (CMV, human herpes virus, parvovirus B19) but may also include other causes (e.g., TMA, hemophagocytic syndrome) [13]. However, an overt SAA meeting Camitta criteria [15] and leading to irreversible bone marrow damage is rare, especially in childhood.

The differential diagnosis of SAA includes viral infections, drug toxicity, cytopenia in the context of hemophagocytosis lymphohistiocytosis (HLH), nutritional deficiencies, myelodysplastic and rheumatologic disorders, and paroxysmal nocturnal hemoglobinuria, along with various congenital causes [16]. The diagnostic work‐up in our patients revealed positive EBV and Parvovirus B19 infections, both known to be associated with SAA [17, 18, 19, 20]. Although the acute clinical presentation with viral‐like symptoms and fever points toward a potential infectious trigger of SAA, the cumulative effect of concomitant multiple medications cannot be excluded. Both tacrolimus and the anticonvulsants (lamotrigine and ethosuximide) that our patient was receiving have the potential to suppress hematopoiesis [21, 22, 23, 24, 25] and could have contributed to severe myelotoxicity. In addition, viral‐induced excessive inflammation given previous long‐lasting immune suppression could have facilitated irreversible damage to hematopoiesis. In patients with SAA younger than 20, an allogeneic HSCT from an HLA‐identical sibling donor or MUD is recommended as a first‐line therapy [6, 26, 27] with cure rates approaching 90% [6, 7, 28]. Immunosuppressive treatment (IST) based on a combination of CSA and anti‐thymocyte globulin (ATG) is an alternative first‐line approach in older patients without an HLA‐identical sibling donor. In the context of prior kidney transplantation, the decision between the most suitable treatment options for SAA is more challenging, requiring leveraging between the desired outcome and potential toxicities. Considering the potential transplant‐related toxicity of an allogeneic HSCT, especially nephrotoxicity, we considered IST as a potential option. Although for patients aged < 20 years the 15‐year overall survival rate reaches 89% in the IST group, response to ATG is delayed, starting after an average of 3–4 months, and reaches only 23% in very severe pancytopenia [27]. It is worth noting that failure to respond to IST implies a rescue HSCT with a higher risk of complications such as acute GVHD grade II‐IV of 25% and chronic GVHD of 20%, respectively [26]. However, as favorable outcomes in children who received kidney and hematopoietic grafts were reported in at least two cases [8], after careful consideration and discussion with the family, we undertook the risk of proceeding with an allogeneic HSCT from a MUD.

One of the largest reports to date regarding the outcomes of HSCT in patients after SOT comes from a retrospective survey of the Japan Society for Hematopoietic Stem Cell Transplantation centres that identified 16 adult patients receiving allogeneic HSCT (7 with liver and 9 with kidney transplants) [1]. Acute leukemia and SAA were the most frequent indications for an allogeneic stem cell graft. The survey reported that all except one patient achieved neutrophil engraftment, as did our patient, but the 5‐year overall survival was poor, reaching (41.7%) and worse in patients with malignancies. Although most of the patients in this report received calcineurin inhibitors and steroids for aGvHD prophylaxis, similar to our case, aGvHD was observed in one‐third of the cases, and most patients after kidney transplantation experienced graft dysfunction. Importantly, none of the patients with severe CMV infection or TMA were reported.

Our patients experienced three major HSCT‐related toxicities that severely complicated the post‐transplant course, including acute GvHD, refractory CMV infection, and TA‐TMA. The patient initially developed acute GvHD that required escalation of immunosuppressive therapy, which significantly worsened pre‐existing hypertension and led to treatment‐resistant CMV infection, eventually contributing to the decline in kidney graft function, as reported in other studies [1, 2]. Although international guidelines recommend using alternative antivirals such as foscavir or cidofovir as second‐line therapy for refractory CMV infection, both medications are highly nephrotoxic [29, 30]. Therefore, a decision to use adoptive therapy with CMV‐specific third‐party donor CTL was made for second‐line therapy as soon as the improved GvHD allowed for weaning off steroids. CTL infusion induces clinical or virological response rates between 70% and 86% [31]; however, in our case, it failed to overcome refractory CMV infection. It is also well known that refractory CMV infection increases the risk of development of CMV disease and mortality after HSCT [32, 33].

Ultimately, the patient developed TA‐TMA, which is reported to significantly compromise overall post‐transplant survival [34, 35]. TA‐TMA is a challenging diagnosis, and a diverse range of diagnostic criteria and definitions have been proposed in the past. Recently, a harmonized definition of TA‐TMA was proposed that requires the occurrence of at least four out of seven TA‐TMA features within 14 days [36]. Our patient exhibited six out of the seven proposed features (except for elevated LDH) and also had features associated with high‐risk TA‐TMA (elevated sC5b‐9, heavy proteinuria, and concurrent CMV infection). Treatment with eculizumab was started immediately after the diagnosis of TA‐TMA with a standard weekly weight‐based dose. Despite the clinical and laboratory response observed following the complement inhibition, the overall outcome was unfavorable. Published data suggest that in high‐risk TMA, more intensive drug‐level adjusted eculizumab dosing (every 72 h) seems to improve 1‐year post‐HSCT survival [35]. It is difficult to specifically tease out the exact cause leading to multiple organ failure in the present case, but the cooccurrence of refractory CMV infection and TA‐TMA were the most likely predisposing factors. It is also important to note that TA‐TMA is an independent risk factor for developing pericardial effusion that substantially compromises overall survival after allogeneic HSCT [14, 37].

In conclusion, despite some reports of favorable outcomes in children who received allogeneic HSCT after kidney transplantation [8], our case report illustrates the numerous challenges that may be encountered in this rare and highly specific patient population. In our experience, many decisions have had to be made in the low or even absence of evidence, requiring the constant balancing of risks of kidney graft failure and HSCT‐related complications. There is a lack of knowledge of dose adjustment of the conditioning regimen and the optimal immunosuppressive regimen after the procedure. Patients undergoing allogeneic HSCT may be at significant risk for transplant rejection due to alloreaction between the allograft and HSCT donor‐derived immune cells, leading to an increased risk of solid transplant dysfunction. The patients may also be at higher risk of HSCT‐related complications, the management of which also requires adjustments for the underlying solid organ transplant. Third, the recipients may not be able to discontinue immunosuppressive therapy to prevent allograft solid organ rejection after allo‐HSCT, which can lead to opportunistic infections. However, there is no alternative strategy for managing fatal and persistent damage of hematopoiesis of hematologic malignancy in SOT recipients. Achievement of immune tolerance by sequential stem cell kidney transplantation from the same donor is an emerging approach to prolong kidney graft survival and reduce the risk of late hematopoietic complications but may not always be feasible [38].

## Author Contributions

G.M.: writing original draft, visualization, review and editing; K.A.: review and editing; G.E.V.: review and editing; A.J.: review and editing, conceptualization, supervision; J.R.: writing, visualization, review and editing, conceptualization, supervision. All authors approved the final version of the manuscript.

## Conflicts of Interest

The authors declare no conflicts of interest.

## Supporting information


Table S1.

## Data Availability

Data sharing is not applicable to this article as no new data were created or analyzed in this study.
