# Supplementary material for: Case Report: Hematopoietic Stem Cell Transplantation to Treat Severe Acquired Aplastic Anemia in a Pediatric Kidney Transplant Recipient
Source: Pediatr Transplant. 2025 May 30;29(5):e70108. doi: 10.1111/petr.70108 (PMC12124711; doi:10.1111/petr.70108)
Supplement: Supplementary file 1 — Table S1. [file PETR-29-e70108-s001.docx]

**Supplemental Table 1.** Post-transplant immune reconstitution on day +55.

|  | Patient value | Institutional age-adjusted reference range |
| --- | --- | --- |
| Total lymphocyte count (cells/mcL) | 378 | 2000-3700 |
| CD3+ (%) | 50 | 66-76 |
| CD3+ (cells/mcL) | 189 | 1400-2000 |
| CD3+CD4+ (%) | 48 | 33-41 |
| CD3+CD4+ (cells/mcL) | 181 | 700-1100 |
| CD3+CD8+ (%) | 2 | 27-35 |
| CD3+CD8+ (cells/mcL) | 8 | 600-900 |
| CD4/CD8 ratio | 19,6 | 1-2,5 |
| CD19+ (%) | 9 | 7-24 |
| CD19+ (cells/mcL) | 34 | 200-700 |
| CD16/CD56+ (%) | 40 | 9-16 |
| CD16/CD56+ (cells/mcL) | 151 | 200-400 |
